# Paracrine IGF-1 Activates SOD2 Expression and Regulates ROS/p53 Axis in the Treatment of Cardiac Damage in D-Galactose-Induced Aging Rats after Receiving Mesenchymal Stem Cells

**DOI:** 10.3390/jcm11154419

**Published:** 2022-07-29

**Authors:** Wei-Syun Hu, Wei-Yu Liao, Chin-Hsien Chang, Tung-Sheng Chen

**Affiliations:** 1 School of Medicine, College of Medicine, China Medical University, Taichung City 40042, Taiwan; weisyunhu@gmail.com; 2 Division of Cardiovascular Medicine, Department of Medicine, China Medical University Hospital, Taichung City 40447, Taiwan; 3 Traditional Chinese Medicine Department, En Chu Kong Hospital, New Taipei City 40237, Taiwan; 01425@km.eck.org.tw; 4 Department of Cosmetic Science, Chang Gung University of Science and Technology, Taoyuan 33303, Taiwan; 5 College of Chinese Medicine, China Medical University, Taichung City 40402, Taiwan; 6 Graduate Program of Biotechnology and Pharmaceutical Industries, National Taiwan Normal University, Taipei 11677, Taiwan

**Keywords:** cardiac aging, mesenchymal stem cells, paracrine, IGF-1, oxidative stress, antioxidant

## Abstract

Aging is one of the causative agents associated with heart failure. Cell-based therapies show potential in the treatment of cardiac aging due to the characteristics of stem cells, including differentiation and the paracrine effect. This study aimed to investigate in detail the mechanism related to biomolecules released from mesenchymal stem cells in the treatment of cardiac aging. In vitro and in vivo models were designed to explore the above hypothesis. Experimental results from the in vitro model indicated that the elevation of oxidative stress, the expression of aging marker p53, and the suppression of antioxidant marker SOD2 could be found in D-galactose-stressed H9c2 cardiomyoblasts. The co-culture of D-galactose-stressed H9c2 with mesenchymal stem cells significantly improved the above pathological signaling. An animal model revealed that the change in cardiac structure, the accumulation of fibrotic collagen, and the activation of the above pathological signaling could be observed in heart tissues of D-galactose-stressed rats. After the rats had received mesenchymal stem cells, all the pathological conditions were significantly improved in D-galactose-stressed hearts. Further evidence indicated that the release of the survival marker IGF-1 was detected in a stem-cell-conditioned medium. Significant increases in cell viability and the expression of SOD2, as well as a reduction in oxidative stress and the suppression of p53, were found in D-galactose-stressed H9c2 cells cultured with a stem-cell-conditioned medium, whereas the depletion of IGF-1 in stem-cell-conditioned medium diminished the antiaging effect on H9c2 cells. In conclusion, the paracrine release of IGF-1 from mesenchymal stem cells increases the expression of antioxidant marker SOD2, and the expression of SOD2 reduces oxidative stress as well as suppresses p53, leading to a reduction in cardiac senescence in D-galactose-stressed rats.

## 1. Introduction

The prevalence of cardiovascular diseases (CVD) is positively associated with age. In accordance with statistics from The American Heart Association (AHA), the incidence of CVD in American men and women is nearly 40% for those aged 40–59 years, 75% for those aged 60–79 years, and 86% for those above the age of 80 [1]. Thus, aging seems to be one of the causative agents in the progress of cardiomyopathy.

From the literature, it is clear that some pathological modifications are often found in cardiomyopathy, including structural and molecular changes. The progress of cardiomyopathy can activate matrix metalloproteinases (MMPs) and suppress tissue inhibitors of metalloproteinases (TIMPs). The imbalance of MMPs and TIMPs modifies the extracellular matrix (ECM) and changes the cardiac structure, such as through the accumulation of collagen fiber in ECM, resulting in cardiac hypertrophy and fibrosis. Structural changes in cardiomyopathy are proportional to age [2,3,4]. In addition to structural changes, cardiomyopathy induced by aging is also linked to some pathological factors, including oxidative stress, inflammation, and apoptosis. These pathological factors are significantly associated with the induction of molecular changes [1]. Yazdanyar et al. [1] stated that an increase in the production of reactive oxygen species (ROS) is found in aged hearts, and an increase in ROS level can produce proinflammatory markers, including interleukin-6 (IL-6) and tumor necrosis factor-α (TNFα). The induction of proinflammatory markers increases chronic inflammatory responses, leading to the apoptosis of cardiomyocytes [5]. Furthermore, the production of ROS in heart tissues induces the expression of the aging marker p53, resulting in the progress of heart senescence. 

The D-galactose-induced aging model is well-established and well-recognized worldwide for the investigation of animal senescence. D-galactose is a reducing sugar that can react with amino acids to form an unstable Schiff base. The unstable Schiff base compounds are then oxidized to produce stable compounds, such as protein carbonyls and advanced glycation end-products (AGEs) [6]. These oxidized compounds increase ROS production by reacting with membrane proteins (including the receptor of AGE and NADPH oxidase), resulting in aging [7]. The administration of D-galactose (60–150 mg/kg/day) for experimental animals (6–8 weeks) is capable of accelerating the aging process in animals [8]. The elevation of several markers can be observed in D-galactose-induced animal aging, including increased AGEs, suppressed SOD, and elevated p53 [6,7,9]. 

Several therapeutic strategies can reduce the progress of cardiac aging, including caloric restriction and exercise training [10,11]. Among these strategies, stem-cell-based therapy shows potential in the treatment of cardiac aging due to stem cell characteristics, including differentiation, self-renewal, and the paracrine effect [12,13]. Stem cells can be roughly classified into three types, namely, embryonic stem cells (ESCs), inducible pluripotent stem cells (iPSCs), and mesenchymal stem cells (MSCs). Although ESCs and iPSCs show higher stemness than MSCs, some disadvantages restrict the use of ESCs and iPSCs in the treatment of cardiac aging, such as ethical concerns and the risk of tumorigenesis. Thus, MSCs have become one of the most appropriate approaches used in clinical practice [14,15,16,17]. The differentiation ability is one of the characteristics of stem cells in tissue regeneration, such as the differentiation to cardiomyocytes or endothelia [18]. In addition to differentiation, the paracrine effect is another important route for stem cells to repair damaged tissue, including the secretion of chemokines, growth factors, or cytokines. All the factors released from stem cells are called the secretome [19]. Cheng et al. [20] stated that bone marrow stem cells secrete several factors under stress, including IL-6, IL-8, TIMP-2, VEGF, and MCP-1, in order to protect stem cells themselves or perform tissue regeneration. From the above description, although the stem cell secretome shows potential in repairing tissues, few papers have discussed the details of the cellular mechanisms between the stem cell secretome and the regeneration of an aging heart. Thus, this study aimed to explore the pathological signaling related to aging hearts and the therapeutic effect of the stem cell secretome on aging hearts through both cellular and animal models.

## 2. Materials and Methods

### 2.1. Chemicals and Reagents

All the chemicals and reagents used in this study were purchased from Merck (Merck KGaA, Darmstadt, Germany).

### 2.2. Cell Culture

H9c2 cardiomyoblast cells (ATCC CRL-1446, USA) were cultured in Dulbecco’s modified Eagle Medium containing 10% fetal bovine serum (Cytiva, Marlborough, MA, USA) and 1% Antibiotic-Antimycotic under an incubator at 5% CO_2_ and 37 °C. Human Wharton’s jelly stem cells (WJSC, Tseng Hsiang Life Science Ltd., Taipei, Taiwan) were cultured in HiMesoXL Mesenchymal Stem Cell Expansion Medium (HiMedia Laboratories Pvt. Ltd., Thane West, Maharashtra, India) containing 10% fetal bovine serum (Cytiva, Marlborough, MA, USA) and 1% Antibiotic-Antimycotic under an incubator with 5% CO_2_ and 37 °C. When performing the co-culture, a hanging insert (PET, 0.4 μm, 24 wells, Merck KGaA, Darmstadt, Germany) was placed in the culture dish and used to separate the H9c2 cells (in a 10 cm culture dish with 80% confluency) and WJSC (in the insert with 5 × 10^5^ cells) into different layers, and H9c2/WJSC co-culture was performed in Dulbecco’s Modified Eagle Medium containing 10% fetal bovine serum (Cytiva, Marlborough, MA, USA) and 1% Antibiotic–Antimycotic in an incubator at 5% CO_2_ and 37 °C.

### 2.3. Cell Viability Assay

H9c2 cell viability was examined using an MTT (3-[4,5-dimethylthiazol-2-yl]-2,5 diphenyl tetrazolium bromide) assay. Briefly, 8000 H9c2 cells were seeded in a 24-well culture dish for 48 h in the presence of different dosages of D-galactose. Then, the culture dishes were washed with PBS, the PBS was discarded, and 0.5 mL of MTT reagent was transferred for incubation for 4 h. After incubation, 0.5 mL of isopropyl alcohol (IPA) was transferred to each well and mixed for 1 min. Then, 200 μL of MTT reagent/IPA mixture was pipetted to a 96-well plate for the measurement of absorbance at 570 nm. For H9c2/WJSC co-culture, 80% of the H9c2 cells were seeded in a 6-well culture dish with a D-galactose concentration of 10 g/L for 48 h. After incubation for 48 h, a hanging insert with 5 × 10^5^ WJSC was added into a 6-well dish for co-culture for 24 h. After incubation, the MTT assay (as mentioned previously) was performed for the measurement of H9c2 cell viability. For H9c2 culturing with anti-IGF1 antibodies, 8000 H9c2 cells were seeded in a 24-well culture dish for 48 h. The experiment was divided into 4 groups, namely, control (8000 H9c2 cells seeded in a 24-well culture dish with 5 mL basal medium), D-galactose (the control group with the presence of D-galactose with 10 g/L), WJSC-CM (the D-galactose group with 4 mL basal medium and 1 mL WJSC-conditioned medium), and anti-IGF1 antibody (the WJSC-CM group with 20 ng of anti-IGF1 antibodies). After 48 h of incubation, H9c2 cell viability was examined using an MTT assay (mentioned previously).

### 2.4. Thiobarbituric Acid Reactive Substances (TBARS) Analysis

The measurement of oxidative stress in tissue or cell lysate can be performed with TBARS analysis. In this study, TBARS analysis was performed in accordance with the manufacturer’s instructions (Abcam plc., Cambridge, UK). Briefly, an 80% confluency of H9c2 cells was seeded in a 10 cm culture dish for 48 h in the presence of different dosages of D-galactose. After incubation, cell lysate was collected by adding RIPA buffer. Then, a thiobarbituric acid (TBA) solution was mixed with the tissue or cell lysate. The TBA/sample mixture was then incubated in a water bath at 95 °C for an hour. After the water bath, the TBA/sample mixture was cooled to room temperature, and n-butanol was added. The TBA/sample mixture was mixed well and then centrifuged at 1200× *g* for 5 min. The upper layer (n-butanol layer) was then transferred to a 96-well plate, and the optical density was read at the wavelength of 532 nm.

### 2.5. Determination of Protein Carbonyl Content in Heart Tissue

Protein carbonyls are stable oxidized compounds, and the content of protein carbonyls is proportional to the level of oxidative stress. The protein carbonyl content in the tissues was determined using a commercial kit (Protein Carbonyl Content Assay Kit, Merck KGaA, Darmstadt, Germany). Briefly, an 80% confluency of H9c2 cells was seeded in a 10-cm culture dish for 48 h in the presence of different dosages of D-galactose. After incubation, cell lysate was collected by adding RIPA buffer. Then, the cell lysate or tissue samples reacted with 2,4-dinitrophenylhydrazine (DNPH) to form stable dinitrophenyl (DNP) hydrazone adducts. Then, the samples were measured spectrophotometrically with a wavelength of 375 nm. The optical density is proportional to the level of protein carbonyl content.

### 2.6. Western Blot Analysis

The expression of target proteins can be quantified using Western blot analysis. Briefly, an 80% confluency of H9c2 cells was seeded in a 10 cm culture dish for 48 h in the presence of different dosages of D-galactose. After incubation, the cell lysate was collected by adding RIPA buffer. Then, the cell lysate or homogenized cardiac tissues were placed on SDS separation gel with a constant voltage (75 V). After separation, the SDS gels were covered with polyvinylidene difluoride (PVDF) membranes with a constant voltage (50 V) for protein transfer. Next, the PVDF membranes were placed in Tris buffer containing 3% bovine serum albumin. Then, the PVDF membrane was placed in solutions containing the primary (p53, SOD2, and GAPDH antibodies were purchased from Santa Cruz Biotechnology, Texas, USA) and secondary antibodies. Finally, the proteins were visualized as blotting bands under a fluorescent detector (Fujifilm LAS-3000, GE Healthcare). Software ImageJ can be applied as a quantified tool to determine the intensity of blotting bands.

### 2.7. IGF-1 ELISA Analysis

An enzyme-linked immunosorbent assay (ELISA) can be applied for the determination of IGF-1 concentration in the medium. Briefly, WJSC was seeded in a 10 cm culture dish with an 80% confluency for 12 and 24 h. The WJSC cultured for 12 h served as a basal medium, and WJSC cultured for 24 h served as a WJSC-conditioned medium. The stem-cell-conditioned medium and the basal medium were transferred into a 96-well plate and incubated in the wells in accordance with the manufacturer’s protocol (Abcam plc., Cambridge, UK). After incubation, each well was washed with PBS and biotinylated IGF-1 antibodies were added to each well. Then, each well was washed with PBS, and HRP-conjugated streptavidin was added to each well. Finally, each well was washed with PBS, and TMB substrate solution was added to each well in order to enhance color development. All the wells were read for optical density using an ELISA reader with a 450 nm wavelength.

### 2.8. Animal Model

Male 8-week-old Wistar rats (BioLASCO Taiwan Co., Ltd., Taipei, Taiwan) were randomly divided into three groups (*n* = 6), namely, Sham, Aging (D-galactose 200 mg/100 g BW per day, via intraperitoneal injection), and Aging + WJSC (with aging rats receiving 1 × 10^6^ stem cells per rat via tail vein). All the experimental rats were adapted in the animal room with a 12 h light-dark cycle, a 25 °C ambient temperature, and standard chow (Lab Diet 5001, PMI Nutrition International Inc., Brentwood, MO, USA). Aging rats were injected with WJSC when they were found to have a heart disorder, as confirmed by a cardiac echo. After WJSC treatment for 8 weeks, all the rats were sacrificed, and the hearts were isolated for further studies. The animal study was approved by animal IRB (Institutional Animal Care and Use Committee of National Taiwan Normal University, protocol number 108021), and the authors of this study can confirm that the animal model followed the rules set out in the Declaration of Helsinki.

### 2.9. Hematoxylin and Eosin (HE) Stain

Structural changes in animal tissue can be observed through HE staining. Briefly, tissue slices were dewaxed using xylene and rehydrated with distilled water. Next, the slices were placed into a Mayers hematoxylin solution, then placed into distilled water. The distilled water was exchanged until the water was clean. After staining, the slices were transferred to an eosin solution, then placed into distilled water. The distilled water was exchanged until the water was clean. Finally, the slices were dehydrated with ethanol and cleared with xylene, and a coverslip was mounted on them to make them ready for observation.

### 2.10. Masson’s Trichrome Stain

Masson’s trichrome stain was used to detect the accumulation of collagen fiber in the tissue. Basically, the tissue slices were incubated with ethanol/distilled water in order to dewax and rehydrate them. Then, the slices were placed in Weigert’s iron hematoxylin working solution and washed with distilled water. Next, the tissue slices were placed in Biebrich scarlet-acid fuchsin solution and then rinsed with distilled water. All the slices were transferred to phosphomolybdic–phosphotungstic acid solution and then aniline blue solution. Finally, the tissue slices were rinsed with distilled water, incubated in a 1% acetic acid solution, and dehydrated with ethanol. All the stained tissue slices were observed under a microscope to examine collagen accumulation.

### 2.11. Immunohistochemical (IHC) Analysis

Animal heart tissue slices were prepared for IHC analysis. Briefly, all the slices were dewaxed using xylene solution and rehydrated with ethanol/PBS solution. Then, antigen retrieval was performed on the slices using 0.1% trypsin/PBS solution. Once antigen retrieval was completed, the tissue slices were conjugated first with primary antibodies (p53 and SOD2 antibodies were purchased from Santa Cruz Biotechnology, Dallas, TX, USA), then with secondary antibodies. After antibody conjugation, the slices were colored and observed using a microscope. 

### 2.12. Statistical Analysis

All the experiments were performed in triplicate, and the data were expressed as mean ± SD. One-way ANOVA was used to calculate significance for multiple comparisons. A paired T-test was applied to calculate the significance of paired comparisons. Statistical significance can be considered at the level of *p* < 0.05.

## 3. Results

### 3.1. Investigation of H9c2 Cells in the Presence of D-Galactose

**Figure 1:**
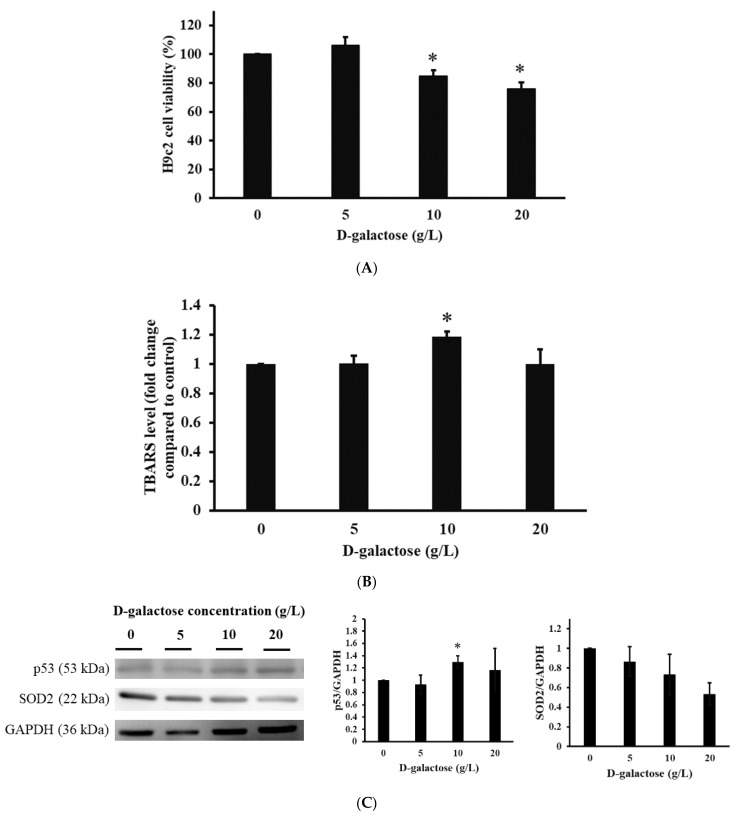
Investigation of H9c2 cardiomyoblast cells in the presence of D-galactose. (**A**) H9c2 cell viability. (**B**) H9c2 TBARS. (**C**) H9c2 protein expression. * *p* < 0.05 compared to 0.

Figure 1 illustrates H9c2 cell damage induced by D-galactose, including cell viability, the level of oxidative stress, and protein expression. From Figure 1A, we can find that cell viability for H9c2 under different dosages of D-galactose followed the order of 100 ± 0, 106.3 ± 5.5, 85 ± 4, and 76 ± 4.6%, respectively. In addition, significance can be observed in 0 vs. 10 (*p* < 0.05) and 0 vs. 20 (*p* < 0.05). Figure 1B shows the level of oxidative stress of H9c2 cells under D-galactose. The level of TBARS is proportional to the level of oxidative stress. In the figure, the level of TBARS for H9c2 cells under different dosages of D-galactose is in the order of 1 ± 0-, 1 ± 0.05-, 1.18 ± 0.04-, and 0.99 ± 0.1-fold change, respectively, and significance is observed in 0 vs. 10 (*p* < 0.05). Figure 1C reveals protein expression for H9c2 cells in the presence of D-galactose. We can observe that the expression of the aging marker p53 in H9c2 cells occurred in a dose-dependent manner. By contrast, the expression of the antioxidant marker SOD2 was decreased in a dose-dependent manner.

### 3.2. Investigation of H9c2/WJSC Co-Culture in the Presence of D-Galactose

**Figure 2:**
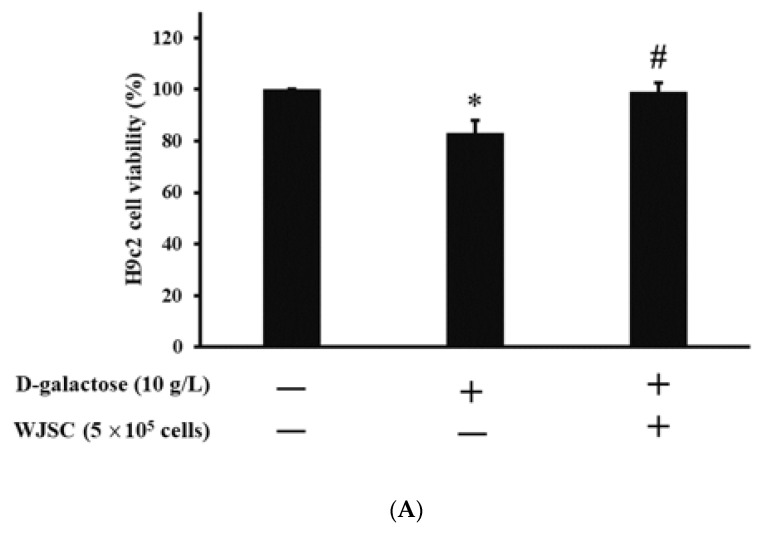

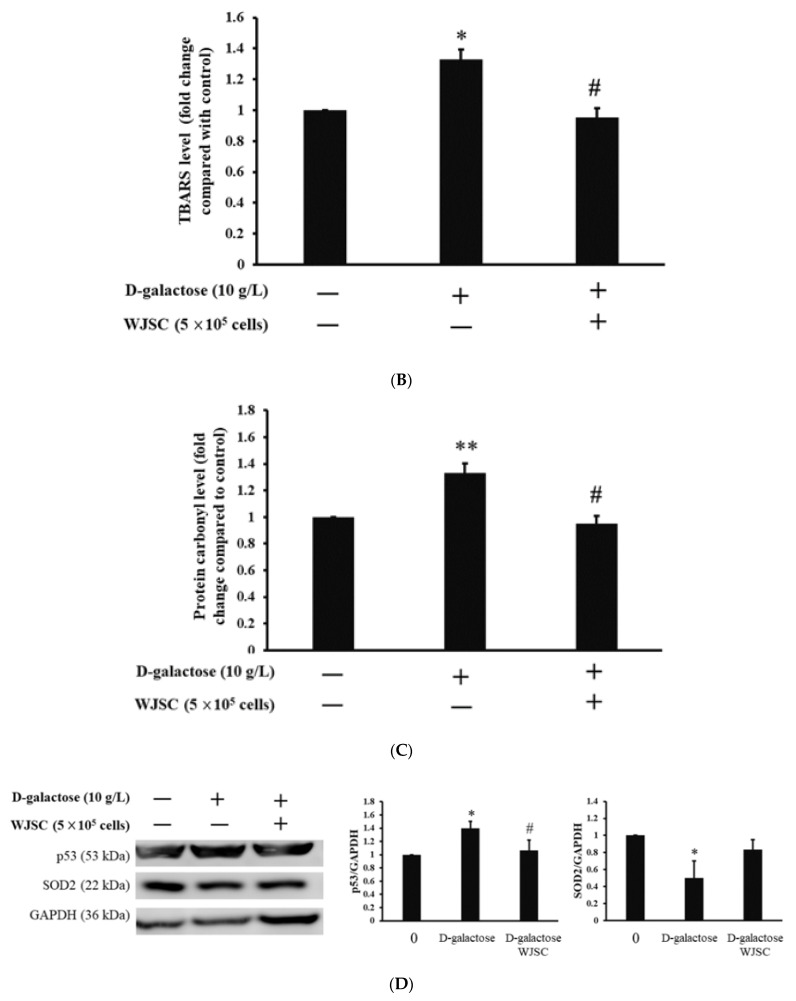
Investigation of H9c2 cardiomyoblast cells co-culturing with WJSC in the presence of D-galactose. (**A**) H9c2 cell viability. (**B**) H9c2 TBARS. (**C**) H9c2 protein carbonyls. (**D**) H9c2 protein expression. * *p* < 0.05 compared to control; ** *p* < 0.01 compared to control; # *p* < 0.05 compared to D-galactose.

Figure 2A shows H9c2 cell viability in the presence of Wharton’s jelly stem cells (WJSC) under D-galactose stress. In the figure, it can be seen that D-galactose significantly reduced H9c2 cell viability (H9c2 > H9c2 + D-galactose, *p* < 0.05). On the other hand, the co-culture of H9c2 cells with WJSC significantly increased H9c2 cell viability in the presence of D-galactose (H9c2 + D-galactose + WJSC > H9c2 + D-galactose, *p* < 0.05). Similar results can be observed in exploring TBARS. In Figure 2B, one can see that D-galactose significantly increased H9c2 TBARS levels (H9c2 > H9c2 + D-galactose, *p* < 0.05). On the other hand, the co-culture of H9c2 cells with WJSC significantly reduced H9c2 TBARS levels in the presence of D-galactose (H9c2 + D-galactose + WJSC > H9c2 + D-galactose, *p* < 0.05). Figure 2C indicates the protein carbonyl content for the H9c2 co-culture with WJSC. Significant observations could be made between H9c2 vs. H9c2 + Aging (*p* < 0.01) and H9c2 + Aging vs. H9c2 + Aging + WJSC (*p* < 0.05). Figure 2D shows the protein expression for H9c2 co-culture with WJSC in the presence of D-galactose. Compared to the control (first column on the left-hand side), D-galactose significantly increased the expression of aging marker p53 (middle column). In contrast, the co-culture of H9c2 with WJSC significantly downregulated p53 expression in the presence of D-galactose (first column on the right-hand side) when compared to the aging group (middle column). An investigation of the antioxidant marker SOD2 was also performed in the H9c2 co-culture with WJSC in the presence of D-galactose. Compared to the control, the D-galactose significantly reduced the expression of the antioxidant marker SOD2 (middle column). Conversely, the co-culture of H9c2 with WJSC significantly increased SOD2 expression in the presence of D-galactose (first column on the right-hand side) when compared to the aging group (middle column).

### 3.3. Investigation of Secreted IGF-1 from WJSC-Conditioned Medium and H9c2 Cell Viability in the Presence of Secreted IGF-1 and IGF-1 Antibodies

**Figure 3:**
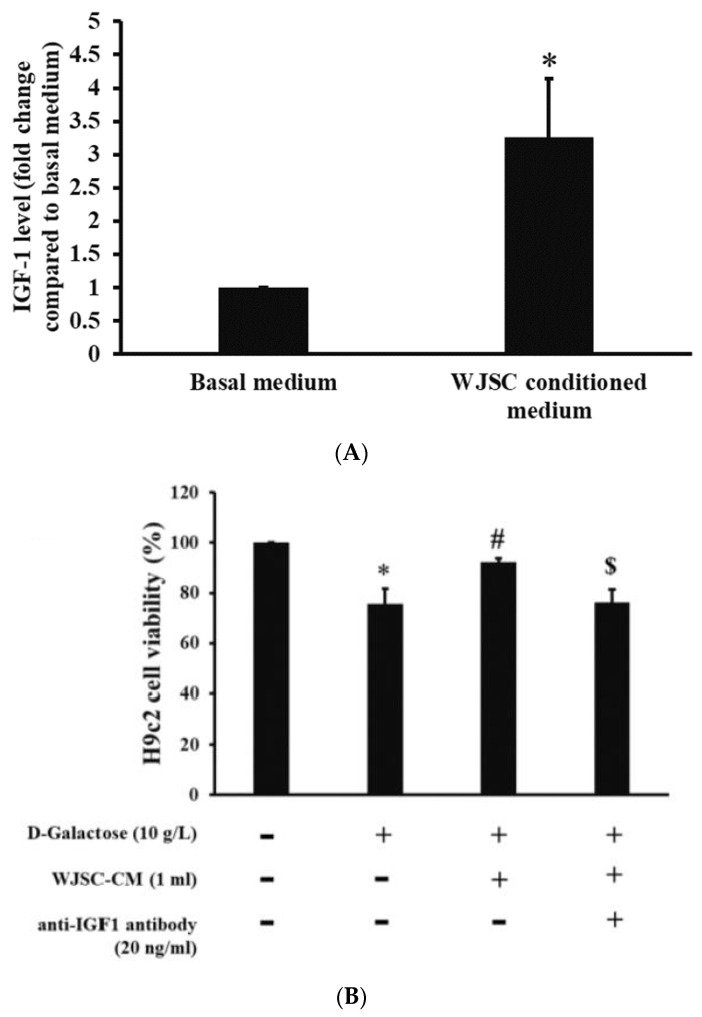
Effect of IGF-1. (**A**) Investigation of IGF-1 expression in the medium. (**B**) Investigation of H9c2 cell viability in the presence of WJSC CM and anti-IGF1 antibody. * *p* < 0.05 compared to control; # *p* < 0.05 compared to D-galactose; $ *p* < 0.05 compared to D-galactose + WJSC-CM.

An enzyme-linked immunosorbent assay (ELISA) can be used to determine protein concentration in the medium. Figure 3A illustrates the amount of change in secreted IGF-1 between basal and WJSC-conditioned media. Compared to the basal medium, there was a significant increase in secreted IFG-1 in the WJSC-conditioned medium (basal medium < WJSC-conditioned medium, *p* < 0.05). Figure 3B indicates H9c2 cell viability in the presence of WJSC-CM (WJSC-conditioned medium) and anti-IGF1 antibodies. Compared to the control (first column on the left-hand side), D-galactose significantly reduced H9c2 cell viability (second column on the left-hand side) (*p* < 0.05). The culture of D-galactose-stressed H9c2 cells with WJSC-CM (third column on the left-hand side) significantly increased cell viability when compared to aging H9c2 cells. Furthermore, a reduction in H9c2 cell viability was found in the culture of D-galactose-stressed H9c2 cells with anti-IGF1 antibodies + WJSC-CM (IGF-1 in the medium neutralized with anti-IGF1 antibodies).

### 3.4. Histological Analysis of Animal Hearts

**Figure 4:**
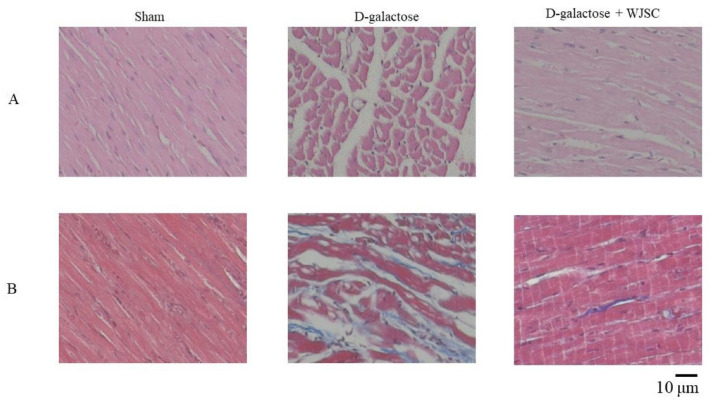
Histological analysis of animal hearts. (**A**) HE staining. (**B**) Masson’s trichrome staining.

Several staining methods can be applied to analyze heart tissue structural changes, including HE staining and Masson’s trichrome stain. Figure 4A shows the HE stain of the experimental heart tissues. Compared to sham, we found that the cardiac structure for D-galactose (D-galactose-stressed animals) became significantly worse, including the disarray of cardiomyocytes and more empty space in the heart tissue. By contrast, significant improvement was found in the aging group receiving WJSC treatment (D-galactose + WJSC). Figure 4B indicates Masson’s trichrome stain for heart tissues. In the tissue, the collagen accumulation was dyed with a blue color, and the increase in the blue area was proportional to the level of tissue fibrosis. Compared to sham, the deposition of collagen (blue color) could be observed in the D-galactose group. On the other hand, a reduction in the blue area in the D-galactose + WJSC group was found compared to the D-galactose group.

### 3.5. Immunohistochemical Analysis of Animal Hearts

**Figure 5:**
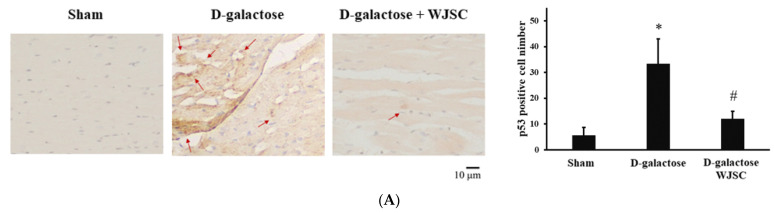

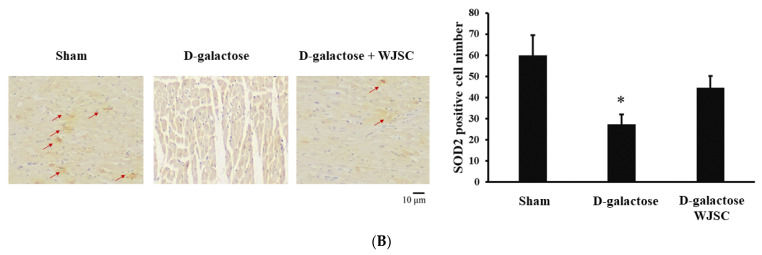
Immunochemical analysis of animal hearts. (**A**) p53. (**B**) SOD2. Red arrows indicate protein expression. * *p* < 0.05 compared to sham; # *p* < 0.05 compared to D-galactose.

Figure 5 shows images of protein expression through the immunochemical analysis of animal hearts. A high expression of aging marker p53 was stained in the D-galactose group (red arrows) compared to sham. By contrast, the suppression of p53 could be observed in the D-galactose + WJSC group compared to the D-galactose group (shown in Figure 5A). Figure 5B reveals the expression of antioxidant marker SOD2 for animal hearts. In the figure, the suppression of SOD2 was found in the D-galactose group compared to sham. On the other hand, the expression of SOD2 was detected in the D-galactose + WJSC group compared to the D-galactose group.

### 3.6. Investigation of Oxidative Marker and Protein Expression in Animal Hearts

**Figure 6:**
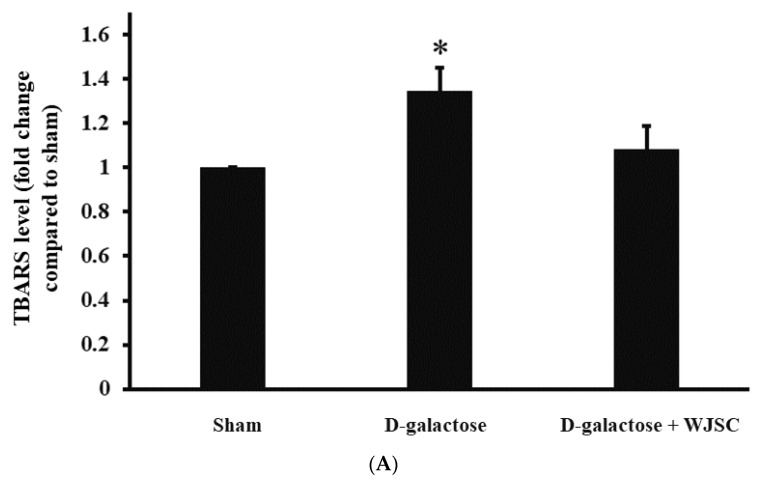

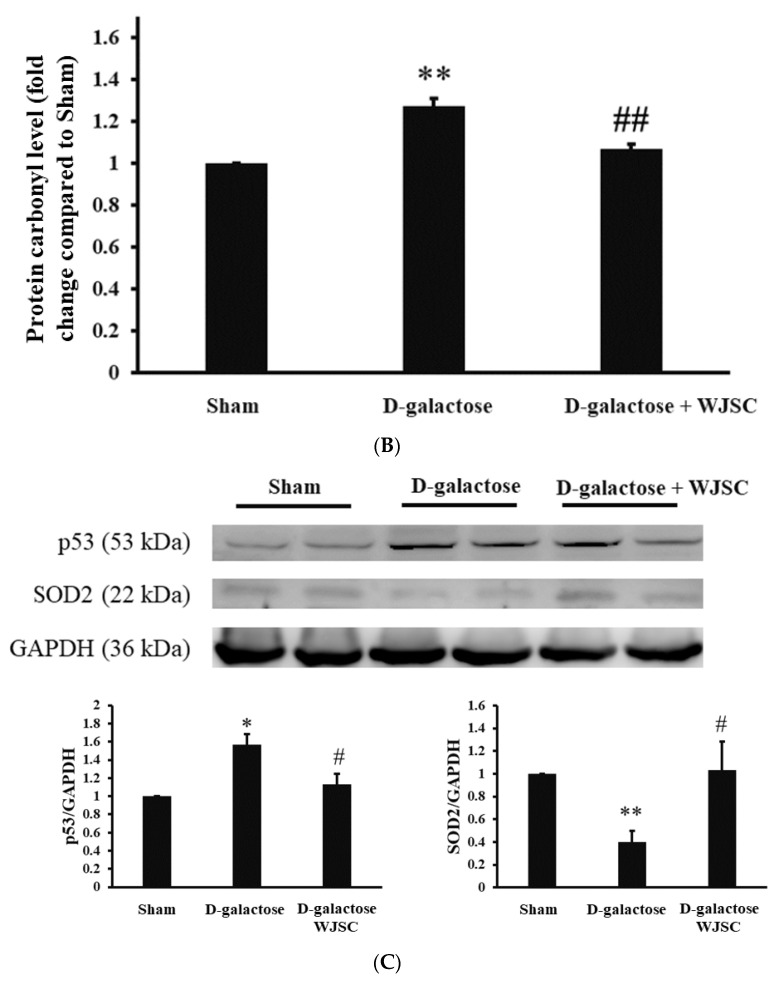
Investigation of animal hearts. (**A**) Heart TBARS. (**B**) Heart protein carbonyls. (**C**) Heart protein expression. * *p* < 0.05 compared to sham; ** *p* < 0.01 compared to sham; # *p* < 0.05 compared to D-galactose; ## *p* < 0.01 compared to D-galactose.

Figure 6A illustrates the levels of TBARS in the animal heart tissues. The TBARS levels for the Aging group were significantly higher than those for the Sham group (Sham < D-galactose, *p* < 0.05). By contrast, the TBARS levels for the D-galactose group were significantly improved in the D-galactose + WJSC group (D-galactose > D-galactose + WJSC, *p* < 0.05). Figure 6B indicates the protein carbonyl content of the heart tissues. Significant observations could be made between sham and D-galactose (*p* < 0.01) and between D-galactose and D-galactose + WJSC (*p* < 0.01). In Figure 6C, the expression of the aging marker p53 in the D-galactose group was significantly higher than in the Sham group. On the other hand, the suppression of p53 in the D-galactose + WJSC group was observed compared to the D-galactose group. For the antioxidant marker SOD2, the suppression of SOD2 was found in the D-galactose group compared to sham. After receiving WJSC, an increase in SOD2 expression was observed in D-galactose + WJSC compared to the D-galactose group.

## 4. Discussion

Structural and molecular changes can be found during cardiac aging, leading to heart failure [21]. Based on histological analysis, HE and Masson’s trichrome staining provided detailed information regarding the structural changes in D-galactose-stressed hearts, including the disarray of cardiomyocytes, enlargement of intercellular space, and the deposition of intercellular collagen (shown in Figure 4). Furthermore, molecular changes could be observed using IHC and Western blot analysis. As mentioned, p53 expression resulted in an increase in oxidative stress and a decrease in the antioxidant marker SOD accompanying the progress of the senescence of heart tissues. The expression of the aging marker p53 and the suppression of the antioxidant marker SOD2 were detected in the D-galactose-stressed hearts (shown in Figure 1, Figure 5 and Figure 6). Furthermore, an increase in oxidative stress was found in both H9c2 cells and heart tissues (shown in Figure 1, Figure 2 and Figure 6). We, therefore, conclude that the aging process increases the production of oxidative stress (the suppression of SOD2 and an increase in TBARS levels along with protein carbonyl content), and the increase in oxidative stress upregulates the expression of the aging marker (the expression of p53) as well as induces tissue remodeling (the disarray of cardiomyocytes, the enlargement of intercellular space, and the deposition of collagen), resulting in heart failure. 

Cell-based therapy shows potential in the treatment of cardiomyopathy induced by aging [22]. In addition to differentiation, the release of biomolecules via the paracrine route (such as proteins or miRNAs) is one of the therapeutic strategies for tissue regeneration triggered by stem cells [23]. Experimental results indicate that a co-culture of D-galactose-stressed H9c2 cells with WJSC and the transplantation of WJSC in D-galactose-stressed rats significantly improves cardiac aging associated with structural and molecular changes in both cell and animal models. As mentioned, the suppression of the antioxidant marker SOD2 and an increase in oxidative stress play a major role in the treatment of cardiomyopathy induced by aging. Gong et al. [24] reported that the blockage of IGF-1 results in the production of ROS due to the depletion of antioxidant biomolecules, including GSH, catalase, and SOD in cardiomyocytes. By contrast, the overexpression of IGF-1 inhibits ROS production due to the expression of antioxidant proteins. These results imply that IGF-1 may act as an upstream signal in the expression of downstream antioxidant proteins. Luo et al. [25] stated that the elevation of ROS is capable of expressing the downstream protein p53, leading to the senescence of hepatocytes. The upregulation of IGF-1 enables the suppression of both ROS and p53, resulting in an antiaging effect on hepatocytes. Thus, the activation of IGF-1 signaling may serve as one of the best policies in the treatment of cellular senescence induced by the ROS/p53 axis. From Figure 3A, we can see significant increases in IGF-1 expression in the WJSC-conditioned medium compared to the basal medium (medium without WJSC precondition). The neutralization of secreted IGF-1 with anti-IGF-1 antibodies in WJSC-CM reduced H9c2 cell viability. This result reveals that the paracrine secretion of IGF-1 from stem cells increases H9c2 cell viability. Furthermore, the co-culture of D-galactose-stressed H9c2 cells with WJSC increased cell viability (Figure 2A), reduced ROS levels (Figure 2B,C), increased the expression of the antioxidant marker SOD2, and suppressed the aging marker p53 (Figure 2D). These experimental findings suggest that the secretion of IGF-1 from WJSC increases the expression of SOD2 in cardiomyocytes, and the expression of SOD2 ameliorates the senescence of cardiomyocytes induced by the ROS/p53 axis.

## 5. Conclusions

**Figure 7:**
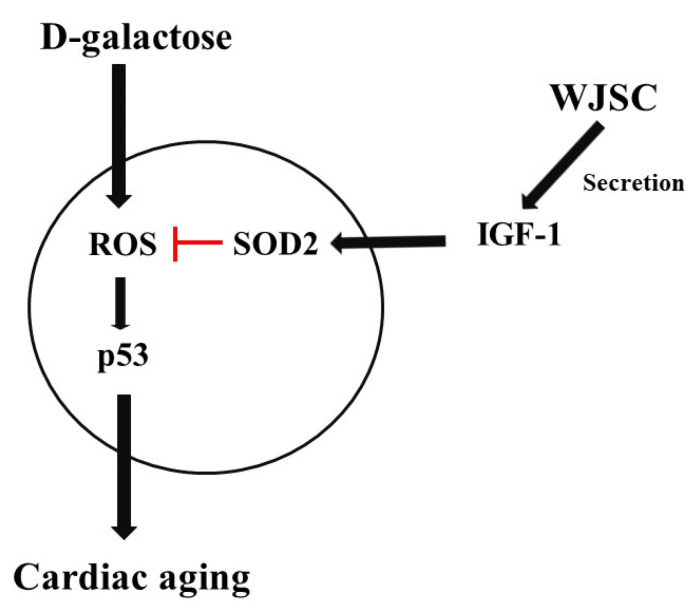
Graphic summary of this study.

The experimental findings can be summarized as follows: (1) structural changes and the activation of the ROS/p53 axis were observed in D-galactose-stressed heart tissues; (2) the secretion of IGF-1 from mesenchymal stem cells increased the expression of the antioxidant marker SOD2 in cardiomyocytes, leading to the inhibition of the progress of cardiomyocyte aging through the inactivation of the ROS/p53 axis. A graphic summary of this study is provided in Figure 7. This study reveals that the secretion of IGF-1-form mesenchymal stem cells via the paracrine route may exhibit therapeutic potential in the treatment of aged hearts.

## Data Availability

All data generated or analyzed during this study are included in the published article.
